# Granulomatosis With Polyangiitis and Concomitant Superinfection: A Defying Diagnosis and Management Approach

**DOI:** 10.7759/cureus.60606

**Published:** 2024-05-19

**Authors:** Fernando Albuquerque, Marcelo Neto, Maria João Cadório, João Oliveira, André Saraiva, Ana Isabel Maduro, Cátia Duarte

**Affiliations:** 1 Rheumatology, Unidade Local de Saúde de Coimbra, Coimbra, PRT

**Keywords:** methotrexate, antineutrophil cytoplasmic antibodies, inflammatory polyarthralgia, rheumatoid factor, granulomatosis with polyangiitis

## Abstract

Granulomatosis with polyangiitis (GPA) is a systemic necrotizing vasculitis mainly involving the ear, nose, and upper and lower airways. Diagnosis is based on clinical manifestations, positive antineutrophil cytoplasmic antibodies (ANCA) serology, and histopathological findings. We report a case of inflammatory polyarthralgia with a high titer of rheumatoid factor (RF), which was revealed to be GPA after extensive diagnosis workup. However, the disease was complicated by superinfections, which delayed and limited immunosuppressive treatment. Methotrexate was at last initiated with antibiotic prophylaxis, and there was significant clinical improvement. This case underlines the importance of an adequate diagnosis workup and the difficulties that often arise when other entities are present.

## Introduction

Granulomatosis with polyangiitis (GPA), formerly known as Wegener's disease, is an antineutrophil cytoplasmic antibody (ANCA)-associated vasculitis (AAV) that predominantly affects small to medium vessels [1,2].

It is a rare disease, with an estimated incidence of 11.8/million person-years and a prevalence of 134.9/million in the United Kingdom, higher in men than women [3]. There are no diagnostic criteria for GPA, and its diagnosis is based on clinical manifestations, positive ANCA serology, and histopathological findings [4].

GPA is a systemic necrotizing vasculitis involving the ear, nose, and upper and lower airways. Life-threatening manifestations, such as alveolar hemorrhage or rapidly progressive glomerulonephritis, may occur. It usually manifests itself as cytoplasmic ANCA-positive (c-ANCA) and/or anti-protease 3 (anti-PR3) antibody-positive [5]. Histopathological findings include necrotizing vasculitis that primarily involves small- and medium-sized vessels, and there may be infiltration of lymphocytes, macrophages, and multinucleated giant cells [6].

After a diagnosis of GPA, the prognosis can vary. Infections are the main cause of early mortality, while advanced renal dysfunction is associated with long-term mortality [5,7]. On the other hand, localized forms with ear, nose, and throat (ENT) involvement have a higher relapse rate [5]. Some studies suggest that rheumatoid factor (RF) positivity may be associated with a different phenotype in AAV patients [8,9].

This article was previously presented as a meeting oral presentation at the XXV Congresso Português de Reumatologia on October 26, 2023.

## Case presentation

We report the case of a 45-year-old female patient who had been suffering for two months from inflammatory polyarthralgia with a migratory pattern affecting the knees, wrists, hands, and feet, with morning stiffness lasting for an hour. The patient was treated with a nonsteroid anti-inflammatory drug with improvement of her symptoms. Additionally, she reported a significant weight loss (more than 5% since the beginning of her complaints), anorexia, and an occasional productive cough. Her past medical history included asthma and allergic rhinitis. She was not taking any medication. There was no history of inflammatory rheumatic diseases in her family.

**Figure 1:**
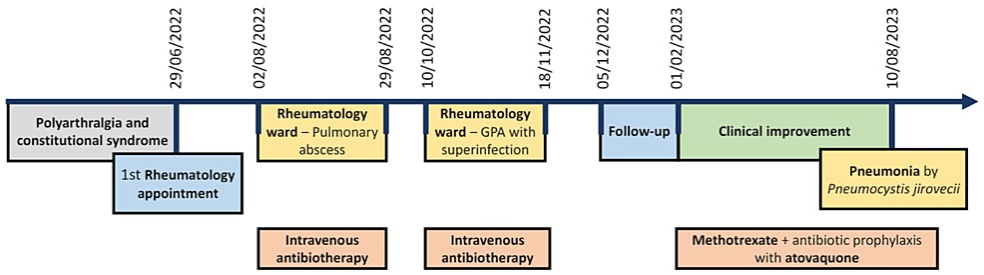
Timeline of events

At clinical examination, the patient presented a body mass index of 18 kg/m^2^. Pulmonary auscultation revealed expiratory crackles in her left hemithorax. She had an antalgic gait due to pain in her feet and ankles. The proximal interphalangeal (PIP) and metatarsophalangeal (MTP) joints, wrists, and ankles were painful on palpation. No joint swelling or other relevant signs were observed. Figure 1 represents the timeline of events.

Laboratory features for diagnostic workup, including hemogram, acute phase reactants, autoimmunity, microorganism cultures, and bacterial and viral serologies, are presented in Table 1.

**Table 1:** Laboratorial findings for diagnostic workup Time 1: first appointment; Time 2: first ward (admission -> discharge); Time 3: second ward (admission -> discharge); Time 4: follow-up (two months after second ward discharge and before starting methotrexate); Time 5: follow-up after two months of methotrexate. ACPA, anti-citrullinate protein antibody; ANA, anti-nuclear antibodies; c-ANCA, cytoplasmic antineutrophil cytoplasmic antibody; anti-PR3, anti-protease 3; IGRA, interferon-gamma release assay; HIV, human immunodeficiency virus; HBV, hepatitis B virus; HCV, hepatitis C virus; CMV, cytomegalovirus; Ig, immunoglobulin; RF, rheumatoid factor

Parameter	Time 1	Time 2	Time 3	Time 4	Time 5	Reference range
Creatinine	0.7	0.6 -> 0.6	0.6 -> 0.7	0.7	0.7	0.6-1.0 mg/dL
CRP	1.8	8.1 -> 0.1	1.2 -> 17.4 -> 1.6	3.0	0.08	<0.5 mg/dL
ESR	41	48 -> 36	38 -> 74 -> 53	44	21	1-20 mm/h
Hemoglobin	11.2	12.6 -> 10.4	9.7 -> 12.0	12.4	12.2	12-16 mg/dL
Leucocytes	16.0	12.7 -> 5.4	12.4 -> 5.3	8.3	8.8	3.9-10.2 G/L
Neutrophils	14.5	9.0 -> 2.7	9.9 -> 3.8	4.1	6.1	1.6-7.7 G/L
Platelets	551	482 -> 291	588 -> 511	401	479	150-400 G/L
RF	445	-	-	-	-	<20 IU/mL
ACPA	<0.04	-	-	-	-	Negative if <7.0 IU/mL; positive if >10.0 IU/mL
ANA	Negative	-	-	-	-	-
c-ANCA	-	-	Strongly positive	Strongly positive	-	-
anti-PR3	-	-	17	25	19	Negative if <2.0 IU/mL; positive if >3.0 IU/mL
Hemoculture	-	Negative	Negative	-	-	-
Sputum culture	-	Staphylococcus aureus	-	-	-	-
Bronchial aspirate culture	-	-	Aspergillus fumigatus	-	-	-
Bronchoalveolar lavage culture	-	-	Negative	-	-	-
IGRA	-	Negative	-	-	-	-
HIV (serology)	-	Negative	-	-	-	-
HBV (serology)	Negative	-	Negative	-	-	-
HCV (serology)	Negative	-	Negative	-	-	-
Syphilisscreening (serology)	-	-	Negative	-	-	-
CMV (serology)	-	-	Previous Infection (IgG positive and IgM negative)	-	-	-
*Chlamydophila pneumoniae* (serology)	-	-	Negative	-	-	-
*Borrelia bugdorferi* (serology)	-	-	Negative	-	-	-
*Coxiella burnetii* (serology)	-	-	Negative	-	-	-
Leptospira (serology)	-	-	Negative	-	-	-
*Legionella pneumophila* (serology)	-	-	Negative	-	-	-
*Mycoplasma pneumoniae* (serology)	-	-	Previous infection (IgG positive and IgM negative)	-	-	-

**Figure 2:**
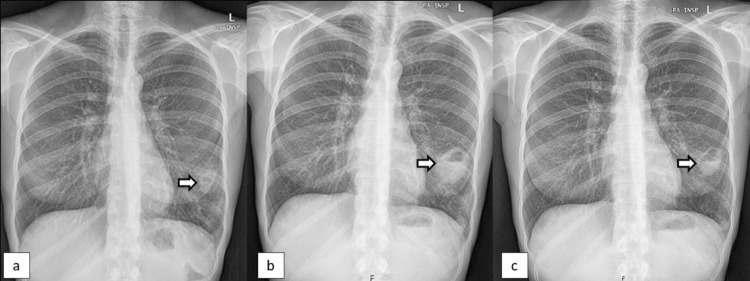
Initial chest X-rays Image (a) reveals a nodule in the left lung of the patient at the first rheumatology appointment. Image (b) shows the evolution of this nodule to a lung abscess, one month later. Image c) shows a reduction of the abscess size after three weeks of intravenous antibiotic therapy.

Blood tests (Table 1 - time 1) showed mild normocytic/normochromic anemia (hemoglobin: 11.2 g/dL), thrombocytosis (platelets: 551 G/L), elevated erythrocyte sedimentation rate (ESR, 41 mm/h), C-reactive protein (CRP, 1.8 mg/dL), negative anti-citrullinate protein antibody (ACPA), and a high level of RF (445 IU/mL; normal range: <20 IU/mL). A musculoskeletal ultrasound was conducted to explore the painful joints, but no signs of synovitis were found. Furthermore, a thoracic radiograph revealed a cavitated nodule in the left lung (Figure 2a). Based on the results, hospitalization was proposed for proper management.

During hospital ward admission, she had a worsening of her cough with sputum. A new thoracic radiograph was performed, which demonstrated an image compatible with a lung abscess in the same location as the previously identified nodule (Figure 2b).

Her blood tests (Table 1 - time 2) revealed an elevation of ESR (48 mm/h) and CRP (8.1 mg/dL), maintaining similar neutrophilic leucocytosis and thrombocytosis. A chest computed tomography (CT) scan confirmed the presence of a lung abscess. Sputum cultures were positive for *Staphylococcus aureus*. The patient was started on antibiotic therapy guided by the results of an antibiogram. The treatment consisted of Linezolid 600 mg administered intravenously every 12 hours, with the addition of Metronidazole 500 mg administered intravenously every eight hours. The patient showed clinical, laboratory, and radiological improvement (Figure 2c). After receiving intravenous antibiotic therapy for three weeks, the patient was discharged.

**Figure 3:**
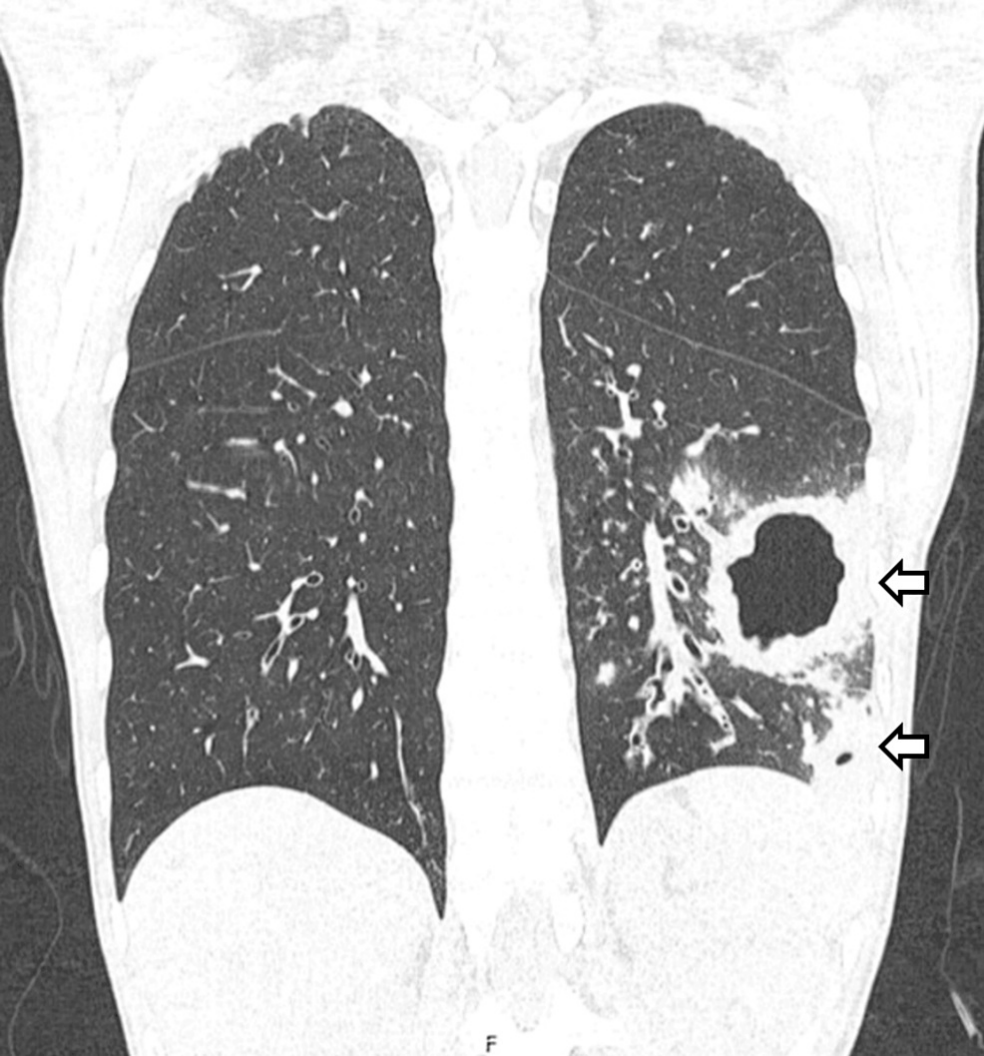
CT scan image (coronal view) one month after hospital discharge CT scan showing three necrotizing masses in the left lung (besides other nodular formations in both lungs) one month after completion of antibiogram-guided antibiotic therapy. CT, computed tomography

One month after being discharged, the patient was readmitted due to a worsening of her musculoskeletal complaints. She was experiencing inflammatory arthralgia with a migratory pattern and new complaints of dysesthesia in her feet. Her ESR and CRP levels were elevated (38 mm/h and 1.2 mg/dL, respectively) (Table 1 - time 3). ANCA testing was performed, and the results were strongly positive for c-ANCA, with anti-PR3 also testing positive (17 IU/mL; normal range: <2 IU/mL; positive if >3 IU/mL). A chest X-ray revealed three nodules in the left lung, confirmed on a CT scan (Figure 3). The CT scan also showed two small nodular images on the right lung.

An echocardiogram and hemocultures were performed, excluding septic embolism. An electromyogram of her lower limbs was conducted, and the test result was negative for mononeuritis or polyneuropathy. A bronchoscopy was carried out, revealing purulent secretions, the culture of which was positive for *Aspergillus fumigatus*. Tests for mycobacterium tuberculosis in the bronchoalveolar lavage and bronchial aspirate, as well as the polymerase chain reaction for mycobacterium tuberculosis complex nucleic acids, were negative. Serological tests for recent viral and other bacterial infections revealed no abnormalities (Table 1 - time 3). The patient received initial treatment for pulmonary aspergillosis with Voriconazole. The treatment consisted of two intravenous doses of 400 mg each, administered at a 12-hour interval, followed by 200 mg orally every 12 hours. Additionally, the patient was given broad-spectrum antibiotic therapy to treat a probable bacterial superinfection. The antibiotic regimen included Piperacillin-Tazobactam 4500 mg intravenously every eight hours and Clindamycin 600 mg intravenously every six hours. At this point, the patient also showed a significant increase in her acute phase reactants (CRP: 17.4 mg/dL; ESR: 74 mm/h). A CT-guided biopsy of the necrotizing mass was performed. Although histology did not identify any preserved lung parenchyma, it identified an intercostal artery with endotheliitis due to T-cells and an extravascular inflammatory infiltrate compatible with GPA.

Gathering all available data, a diagnosis of GPA was established, with active pulmonary involvement and an Aspergillus superinfection. Further investigations were carried out to determine if other organs were affected. Still, there was no evidence of any other vasculitis manifestations, especially regarding ear, nose, and throat (ENT) and renal involvement. The urinalysis and sinus CT were normal, and the evaluation by an otorhinolaryngologist found no abnormalities.

**Figure 4:**
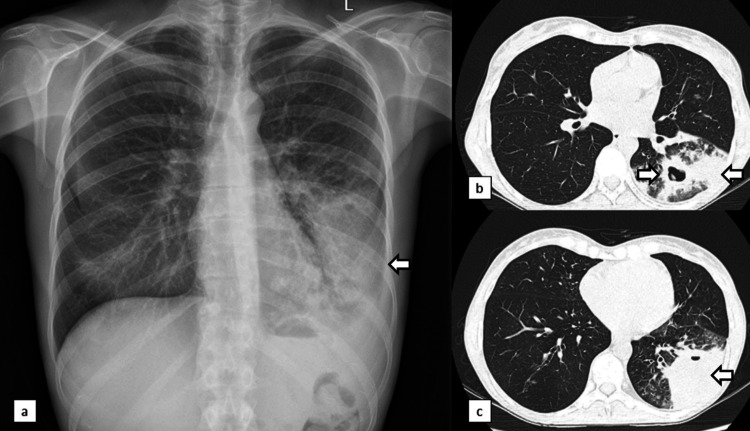
Chest X-rays and CT scan after antibiotic therapy and Voriconazole Chest X-rays (image a) and CT scans (images b and c, axial sections) reveal a complete opacity of the lower lobe of the left lung. CT-guided aspiration was performed, revealing the presence of purulent material. CT, computed tomography

Despite receiving antifungal and broad-spectrum antibiotic treatments, her radiological condition worsened with almost complete opacity of the lower lobe of the lung (Figure 4). The content of the mass was aspirated, revealing the presence of purulent material. However, its culture did not show the presence of any microorganisms.

**Figure 5:**
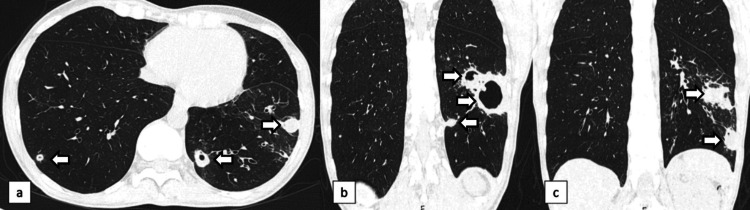
CT scan after four weeks of intravenous antibiotic therapy and Voriconazole CT scan showing cavitated nodules in both lungs, seen in axial (image a) and coronal (images b and c) sections. CT, computed tomography

At four weeks of intravenous antibiotic therapy, there was an improvement in the acute phase reactants and thrombocytosis, although complete resolution was not achieved (Table 1 - time 3). Clinically, the dysesthesia resolved with pregabalin, but she maintained inflammatory arthralgia with a migratory pattern, needing regular nonsteroidal anti-inflammatory drugs. Another CT scan was performed at this point, maintaining some cavitated nodules in both lungs (Figure 5).

After a multidisciplinary discussion, it was decided not to introduce an immunosuppressive drug at this point. The patient was discharged under antifungal (Voriconazole 200 mg orally every 12 hours) and antibiotic therapy (Amoxicillin-Clavulanate 1.2 g orally every 12 hours plus Ciprofloxacin 750 mg orally every 12 hours) for another four weeks in order to fulfill eight weeks of treatment.

Two months after discharge, she maintained a similar clinical and laboratorial status (Table 1 - time 4), with positive anti-PR3 (25 IU/mL). Treatment with methotrexate 15 mg/week was initiated, along with antibiotic prophylaxis with atovaquone 1500 mg taken orally every day. Two months after starting methotrexate, she had a significant clinical improvement, without regular inflammatory arthralgia or other symptoms, and normalization of acute phase reactants (Table 1 - time 5).

**Figure 6:**
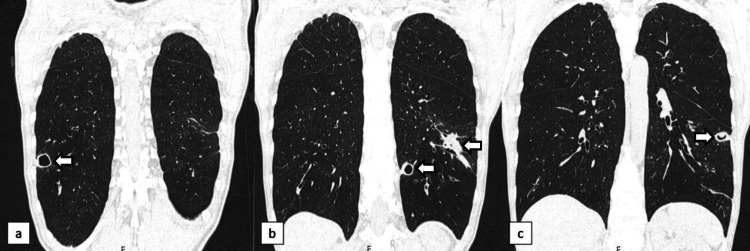
CT scan two weeks after completing antibiotic therapy for pneumocystis pneumonia Images a-c correspond to coronal sections revealing some cavitated nodules with variable dimensions, with typical features of GPA GPA, granulomatosis with polyangiitis

However, six months after initiating methotrexate, she was readmitted due to pneumonia by *Pneumocystis jirovecii*. A Thoracic CT scan was repeated two weeks after completing antibiotic therapy for *Pneumocystis *pneumonia, which revealed some cavitated nodules with variable dimensions, with typical features of GPA (Figure 6). Following the successful resolution of the infection, the patient continued to receive methotrexate and is currently under close monitoring to reassess the activity of the disease. At present, the patient remains asymptomatic.

## Discussion

The presented case was a significant clinical challenge both in its diagnosis and treatment. The first clinical impression suggested an inflammatory arthropathy like rheumatoid arthritis, paraneoplastic syndrome, or an infection. Later, a lung abscess was identified. Even though antibiotic therapy led to clinical improvement, there was a relapse, and three pulmonary masses and nodular lesions on both lungs developed. In this situation, GPA became highly suspicious. However, its diagnosis and management were difficult as there was no evidence of other organ involvement, and the culture of bronchial secretions was positive for *Aspergillus fumigatus*. A CT-guided biopsy of the necrotizing mass with histopathological findings compatible with GPA and positive anti-PR3 supported this diagnosis. Despite adequate treatment for pulmonary aspergillosis and covering other possible microorganisms, there was initial radiological worsening. Aspiration of the greater lung mass revealed a purulent material, but its culture was negative for any microorganism. Even after being on antifungal medication for several weeks associated with broad-spectrum antibiotic therapy, nodular images on thoracic CT scan and slightly elevated acute phase reactants persisted. Thus, the diagnosis of GPA was based on clinical manifestations and evolution, including recurrent infection despite antibiotic therapy, radiological and histopathological features, and positive anti-PR3. A multidisciplinary team was essential to achieve this diagnosis and to manage this difficult case.

GPA diagnosis relies on clinical findings as well as ANCA serology and/or histology findings [4]. When testing ANCA using enzyme-linked immunosorbent assay (ELISA), proteinase 3 (PR3) antigen-specific immunoassay provides a sensitivity of 70-78% and a specificity of 95-97% for GPA [10]. In addition, a meta-analysis revealed a moderate sensitivity and specificity for a positive ANCA as a predictor of AAV relapse [11].

Our patient also presented high titters of RF, making the diagnosis more challenging. In clinical practice, up to 50% of AAV patients have positive RF [9]. The RF is an autoantibody directed against the Fc portion of immunoglobulin G. Despite its association with rheumatoid arthritis, RF positivity can be found in other conditions, such as other connective tissue diseases or infections [12]. On the other hand, the anti-citrullinated protein antibody (ACPA) positivity is rare in GPA, and other features of rheumatoid arthritis, such as bone erosions, are typically absent [13]. In our patient, the absence of polyarthritis, erosions, and ACPA made the diagnosis of rheumatoid arthritis unlikely.

The relevance of RF in AAV has been highlighted in the last few years. Watanabe S. et al. found that RF was associated with higher disease activity assessed by Birmingham Vasculitis Activity Score, higher rate of nervous system involvement, and higher levels of serum CRP and ESR [8]. Recently, Ahn S. et al. reported that AAV patients with positive RF and ANCA presented more frequently with general manifestations and increased markers of inflammation, suggesting that RF and ANCA positivity in AAV patients may be linked to a different disease phenotype with more systemic inflammation. They also reported that end-stage kidney disease-free survival rate was lower in patients with negative RF and positive ANCA [9]. However, in these studies, about one-fourth of the AAVs were GPA. Our patient presented with general symptoms and raised acute phase reactants as well as leucocytosis, neutrophilia, and thrombocytosis, besides high RF titter, matching this distinct phenotype suggested above. Nevertheless, more studies on RF's role in AAV manifestations and prognosis are needed.

Although induction therapy with an immunosuppressive drug such as rituximab combined with corticosteroids is recommended in GPA, a careful benefit/risk analysis had to be made, considering the patient's infectious risk and absence of life-threatening disease [14]. With this in mind, treatment with methotrexate was started without any corticosteroid, with a good clinical and analytic response in the first six months. However, the patient developed a pneumocystosis, and some pulmonary nodules remained in the thorax CT scan. The patient is currently under close follow-up for disease activity reassessment.

## Conclusions

In conclusion, we present a GPA case with constitutional symptoms, inflammatory polyarthralgia, elevated inflammatory serum markers, a high titer of RF, and pulmonary nodules. However, the presence of concomitant superinfection added a significant challenge in both diagnostic workup and management. Positive ANCA, especially anti-PR3, and histopathological examination helped achieve a diagnosis.
